# Flaxseed in Diet: A Comprehensive Look at Pros and Cons

**DOI:** 10.3390/molecules30061335

**Published:** 2025-03-16

**Authors:** Sara Duarte, Muhammad Ajmal Shah, Ana Sanches Silva

**Affiliations:** 1 University of Coimbra, Faculty of Pharmacy, Polo III, Azinhaga de Santa Comba, 3000-548 Coimbra, Portugal; sara.alves.duarte@gmail.com; 2 Department of Pharmacy, Hazara University, Mansehra 21300, Pakistan; ajmalshah@hu.edu.pk; 3 Centre for Animal Science Studies (CECA), Instituto de Ciências e Tecnologias Agrárias e Agro-Alimentares (ICETA), University of Porto, 4501-401 Porto, Portugal; 4 Associate Laboratory for Animal and Veterinary Sciences (Al4AnimalS), 1300-477 Lisbon, Portugal

**Keywords:** flaxseed, diet, benefits, cyanogenic glycosides, determination methods, detoxification methods

## Abstract

Flaxseeds, which have been consumed for thousands of years, have recently gained increasing popularity due to their rich composition, including omega-3 fatty acids, lignans, proteins, and fibers. These components are strongly associated with various health benefits, such as improving cardiovascular health, preventing certain types of cancer, controlling diabetes, promoting gastro-intestinal well-being, and aiding in weight management. This monograph explores the role of flaxseeds in nutrition, as well as their potential risks. Despite their numerous health benefits, flaxseeds also represent concerns due to excessive consumption and possible contamination, particularly from cyanogenic glycosides. Therefore, the levels of these compounds must be controlled, and this monograph also analyzes the available methods to detect and reduce these contaminants, ensuring the safety of flaxseed and flaxseed products consumers. Flaxseed is considered a valuable addition when incorporated into the diet, but it is necessary to continue research and promote technological improvements to maximize their benefits and minimize their risks.

## 1. Introduction

Flaxseed (or linseed, as it is known in British English) is an ancient crop with a rich history, dating back nearly 12,000 years [1]. Originally, flaxseed was likely used for its laxative properties before its widespread consumption as a food ingredient. In recent years, there has been a marked increase in health-oriented products containing flaxseed, driven by growing research that highlights its potential health benefits. Therefore, dietary flaxseed has been the object of a growing research literature supporting its use, as well as trials to help and provide reliable information to the general public, especially to those in a disease-compromised condition [2], but also to the rest of the population who would like to introduce flaxseed in their diet daily. It is increasingly evident that pharmacologic therapy must be complemented by other interventions, such as dietary strategies, to effectively treat diseases or even prevent or delay their onset. Growing awareness of flaxseed’s health benefits is driving increased demand, which is likely to impact farmers, food processors, and retailers in the coming years [2]. However, this rising popularity may also lead to overconsumption, with potential adverse consequences for consumers. In addition, environmental conditions such as heat damage, frosts, and drought can affect the composition and quality of flaxseed. Flaxseed contamination is also possible and can lead to detrimental effects on the consumers’ health, so it is possible contaminants must be known and controlled by safety measures.

Consequently, flaxseed is widely recognized for its health benefits, particularly due to its components, such as alpha-linolenic acid (ALA), proteins, lignans, and fiber. Many reviews focus on its positive effects in disease prevention, treatment, and management, offering insights into its role in promoting human health, which can lead to an oversimplified view of flaxseed’s overall impact and may inadvertently encourage overconsumption, without taking into account the potential risks associated with flaxseed, such as the presence of toxic components.

This paper aims to provide a novel perspective on flaxseed by presenting a comprehensive examination that goes beyond the existing literature. While previous studies have primarily focused on the well-documented health benefits of flaxseed, this work also critically assesses the potential risks associated with its consumption, particularly those arising from improper use, excessive intake, and contamination with cyanogenic glycosides. What sets this study apart is its emphasis on practical, innovative solutions to mitigate these risks, including advanced detection and reduction techniques for toxic compounds, thus, ensuring safer consumption. By integrating these new insights, this article aspires to furnish a more balanced understanding of flaxseed, encompassing both its advantages and drawbacks, ultimately contributing to a more informed perspective on its role in health and nutrition.

## 2. Flaxseeds (*Linum usitatissum* L.)

**Figure 1:**
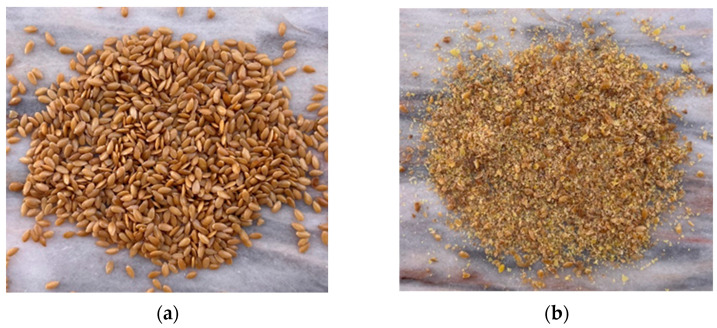
(**a**) Whole golden flaxseed; (**b**) milled golden flaxseed.

Flaxseeds is the common name of *Linum usitatissum* L., also known as common flax when grown for the fiber extracted from its stem, and as linseed or oilseed flax when cultivated for the oil extracted from its seeds. It belongs to the genus *Linum* L. and the family *Linaceae*. Despite the numerous species in this family, flax is the only one cultivated. Flax is an erect, herbaceous annual plant that branches above the main stem [3]. Cultivated flax reproduces by its seed. Because of its perfect flower structure and the composition of its pollen, it is rarely transferred by insects, which makes flax a highly self-pollinated species. Its leaves are simple, sessile, linear-lanceolate with entire margins, borne on stems and branches. Flowers are borne on long erect pedicels, are hermaphroditic, hypogynous, and are composed of five sepals, five petals (blue, pink, white), five stamens, and a compound pistil or five carpels, each separated by a false septum. The fruit is a capsule with five carpels and may contain up to ten seeds. The flax seeds are formed by the seed and the shell, which are oval and flat, lenticular, and 4–6 mm long with pointed tips. The seed contains lignans, digestible proteins, oil rich in omega-3 fatty acids, and phenolics [3]. The shell comprises high-quality fiber with low density and mechanical qualities and holds a percentage of mucilage. It is smooth and glossy and shows a specific color from brown to dark gold [4] (Figure 1).

Flax has been an integral part of human culture for thousands of years, with its origins likely tracing back to the regions east of the Mediterranean, extending toward India. From there, it gradually spread across Asia and Europe before being introduced to the Americas. The domestication of flax is believed to have first occurred in the Fertile Crescent, a region known for its early advancements in agriculture [5]. The use of the crop spread in Europe 5000 years ago and domesticated flax was also cultivated in China and India [3]. The flax industry spread with European powers’ colonization of the New World and other regions [6]. According to the Centre for Agriculture and Biosciences International (2018), flax is grown commercially mainly in Western (France, Belgium, the Netherlands) and Eastern Europe (Russia, Ukraine, Belarus, Poland), the latter has the largest land areas dedicated to flax, but with the best agronomic yields and processing technology in the former. The latest data of FAOSTAT, corresponding to the year 2022, confirms that Europe is the continent with the major production share of flax, i.e., 95.1% of total production. The top three producers worldwide are European countries, which are France, Belgium, and Belarus [7].

Flax thrives in moderate to cool climates, primarily in northern latitudes that receive 150 to 200 mm of rainfall during the main growing season (April–June). It grows best in fertile, well-drained soils with a medium to heavy texture and a pH range of 5.5 to 7.0 [6]. In addition to cultivated fields, flax can also be found in disturbed areas, along roadsides, and in abandoned homesteads at elevations ranging from 0 to 2400 m [3]. Its life cycle consists of a 45–60-day vegetative phase, followed by a 15–25-day flowering period, a reproductive phase, and a maturation stage lasting 30–40 days [3].

Currently, flax is used for food, feed, and industrial purposes. Focusing on the seeds, many food products are manufactured from them, including bread, cereals, crackers, energy bars, omega-3 eggs, and pasta [3]. Flaxseed can change quality factors like moisture, color, and hardness, which can significantly influence consumer acceptance of a product [8]. For people with celiac disease or gluten sensibility, flaxseed powder is a substitute for baking ingredients; in addition, results show that adding flaxseed powder enhances the number of calories, protein, ash, acidity, and antioxidant qualities [9]. Besides its use as seed for planting, the primary industrial application of flaxseed is processing it to produce flaxseed oil and meal. Due to its high protein content, a byproduct of flaxseed can be used as a supplement in livestock, including poultry feed. Linseed oil is highly valued in paints and varnishes due to its exceptional drying properties, which stem from its unique fatty acid composition. This characteristic makes it an essential ingredient in protective coatings and artistic applications. Additionally, the plant’s stem fibers are processed to produce high-quality linen textiles and fine paper, further showcasing the versatility and economic importance of flax [3].

Although this section addresses the presence of flax and its seeds in various contexts, this paper will specifically focus on the composition of flaxseeds and their potential benefits and drawbacks for human health.

## 3. Composition of Flaxseed

Flaxseeds are primarily categorized into two types: yellow (or golden) flaxseeds and brown flaxseeds. The main difference between them lies in their color and potential nutrient profiles, although both types are rich in omega-3 fatty acids, lignans, and dietary fiber. Yellow flaxseeds tend to have a slightly milder flavor and are often favored by those looking for a more neutral taste in recipes. Brown flaxseeds, on the other hand, are more commonly found in grocery stores and have a more robust, nutty flavor. Some studies suggest that there may be slight variations in their nutritional content, with brown flaxseeds potentially boasting a higher lignan concentration [10]. However, both varieties offer similar health benefits, making them interchangeable in most culinary uses. Additionally, there are specialty flaxseed varieties, such as high-lignan flaxseeds, bred for increased health benefits, which can further diversify options for consumers.

### 3.1. Nutrients

According to the Portuguese Food Composition Database (INSA-National Health Institute Dr. Ricardo Jorge), 100 g of raw flaxseed contains 487 kcal. The energetic distribution is 56.7% lipids, 21.0% protein, 15.2% carbohydrates, and 7.1% fiber. The corresponding composition includes 100 g of raw flaxseed, 31 g of lipids, 25 g of protein, 18.1 g of carbohydrates, 18 g of fiber, 4.9 g of water, and 3.0 g of others (inorganic compounds) [10].

The lipids fraction (per 100 g) comprehends 2.7 g of saturated fatty acids, 5.5 g of monounsaturated fatty acids, and 21 g of polyunsaturated fatty acids, 6.3 g of which is linoleic acid. Sugars, sucrose, and starch constitute the carbohydrates in the following amounts: 5.2 g, 2.7 g, and 10.7 g, per 100 g, respectively [11].

Other studies have reported different values and variations in composition, which may result in slight discrepancies among studies. The natural variability in components arises from several factors, including differences in geographic origin, soil quality, climate conditions, agricultural practices, and seasonal variations [3,4]. These factors can influence the nutrient composition, bioactive compounds, and overall quality of food, leading to variability in the results of food analysis. Research has shown that factors such as heat damage can lead to alterations in oil yield and fatty acid profiles, while frost events can negatively affect seed viability and nutrient content [12,13]. Additionally, prolonged drought conditions have been linked to reduced seed weight and overall crop health. For example, studies by Melelli et al. [12] and Zare et al. [13] highlight the detrimental effects of extreme temperatures and water stress on flaxseed quality. By integrating these findings, it becomes evident that understanding and mitigating the impacts of environmental stresses is vital for optimizing flaxseed production and ensuring product quality.

The sections below provide a more detailed description of the key constituents of flaxseed and explain why they contribute significantly to its value.

#### 3.1.1. Alpha-Linolenic Acid

Alpha-linolenic acid (ALA) is a component of flaxseed oil, which contains a mixture of mono- and polyunsaturated, as well as saturated, fatty acids. The unsaturated fraction is the largest (87.8 to 89.8%), with ALA representing the majority of it [14], according to a study carried out by Qiu et al., while 47.44–53.67% of flaxseed oil is formed by linolenic acid, 19.56–24.33% by oleic acid, 12.8–15.01% by linoleic acid, 5.01–9.57% by stearic acid, and 5.14–6.43% by palmitic acid [15].

ALA is an essential omega-3 fatty acid that serves as a precursor to eicosapentaenoic acid (EPA) and docosahexaenoic acid (DHA), both of which are linked to various health benefits. Since the human body cannot synthesize ALA, it must be obtained through diet. While seafood, particularly fatty fish, is the primary source of EPA and DHA, these beneficial omega-3s are also present in oilseeds like flaxseed. Epidemiological studies and randomized controlled trials consistently highlight the positive impact of omega-3 polyunsaturated fatty acids (PUFAs), particularly EPA and DHA, on long-term health. These benefits include reduced cardiovascular disease morbidity and mortality, improved visual and neurological development, and better outcomes in inflammatory conditions such as arthritis and asthma. However, the conversion of ALA to EPA and DHA in the body is limited in humans, with the conversion of ALA to EPA estimated at 8–12% and to DHA less than 1%. The efficiency of this conversion may vary by gender, with women typically exhibiting higher conversion rates than men. Research has reported that in men, the conversion of ALA to EPA ranges from 0.3% to 8%, whereas in women, it can be as high as 21%. Similarly, the conversion of ALA to DHA is less than 1% in men, but in women, this efficiency can reach up to 9%. This highlights the need for continued research into factors that influence this conversion and the potential health benefits of ALA supplementation and, consequently, diets including flaxseed, given its clear promise for delivering significant health advantages [16].

#### 3.1.2. Proteins

Flaxseed is a plenteous protein source, representing 23% of the overall seed mass, increasing to 35 to 40% after oil extraction. The protein provided by flaxseed is complete, meaning that all the essential amino acids are present in adequate amounts and its consumption can overcome protein deficiency. One of the latest studies revealed which amino acids are present in high content in flaxseed plants; this diversity includes arginine, alanine, glycine, isoleucine, histidine, lysine, and leucine [17]. The sulfur-based amino acids, cysteine, and methionine are also present in significant amounts, and when compared to other plenteous proteins, such as soy, flaxseed can be a preferable choice as an animal protein substitute, for drug formulations and infant formula, for example. Furthermore, proteins play a crucial role in cardiovascular health by helping to reduce cholesterol levels, thereby exerting a positive impact on cardiovascular disease management [4].

#### 3.1.3. Fiber

Flaxseed has been found to have up to 40% dietary fiber in its composition, 25% of which is insoluble fiber majorly represented by cellulose, lignin, and hemicellulose [4]. The remaining 75% is the portion of soluble fiber that includes gums, pectin, and β-glucan. The dietary fiber in flaxseed can lower blood sugar levels, absorb cholesterol and triglycerides, enhance serum lipid profiles, and raise basal metabolism [4]. These effects of its composition in fiber can have an important role in the prevention of diabetes and cardiovascular disease. Moreover, flaxseed, as a source of soluble fiber, constitutes a dietary source for bacteria in the gut, consequently enhancing gut flora and improving gastrointestinal health [4].

#### 3.1.4. Vitamins

Flaxseed contains both water (ascorbic acid, thiamine, and riboflavin and vitamin C, B1, and B2, respectively) and fat-soluble vitamins. The last ones, although in small amounts (mg/100 g), are present in greater quantities than the first ones. According to the Portuguese Table of Food Composition, raw flaxseed (per 100 g) has in its composition different vitamins, including α-tocopherol (8.2 mg), thiamine (0.79 mg), riboflavin (0.25 mg), niacin (7.9 mg), vitamin B6 (0.79 mg), and folates (97 μg) [11].

The fat-soluble vitamins in flaxseed are represented by the abundant vitamin E (569 mg/100 g), fundamentally, as γ-tocopherol (552 mg/100 g), but also α-tocopherol (7 mg/100 g) and δ-tocopherol (10 mg/100 g), a known antioxidant that protects cell proteins and fats from oxidation, regulates sodium elimination within the urine, which might assist lowering its blood load, which is important in heart disease or its prevention. Vitamin K, a basic component in the blood-clotting mechanism, is also present in flaxseed but only when it is milled (0.3 mg/tablespoon) [4].

#### 3.1.5. Minerals

Minerals are essential in human nutrition since they regulate various metabolic processes and their importance in immunity, growth, and development. Flaxseed is not only a rich source of macronutrients (carbohydrates, proteins, and fats) but also micronutrients, which are vitamins and minerals. Magnesium, potassium, phosphorus, and sodium are the macro-minerals present in flaxseed, while zinc, copper, and iron are the most relevant micro-nutrients. A recent study by Noreen et al. gave values for some of the minerals present in flaxseed: potassium (763.7 mg/100 g), phosphorus (581.5 mg/100 g), magnesium (406.6 mg/100 g), iron (5.13 mg/100 g), and zinc (3.30 mg/100 g) [17].

According to the Portuguese Table of Food Composition, the inorganic compounds of flaxseed (per 100 g) are sodium (11 mg), potassium (470 mg), calcium (200 mg), phosphorus (550 mg), magnesium (350 mg), iron (15 mg), zinc (7.8 mg), and selenium (28 μg) [11].

### 3.2. Phenolic Compounds

Phenolic compounds can be categorized into several main classes: phenolic acids, flavonoids, and lignans.

#### 3.2.1. Lignans

Flaxseed is the richest plant-based source of lignans, providing up to 0.7–1.5% of its dry weight in these compounds, which are highly beneficial to human health [18]. Lignans present in flaxseed are essentially mataresinol, pinoresinol, diphyllin, (in minor quantities), and secoisolarisericinol [16]. The primary lignan in flaxseed is secoisolariciresinol diglycoside (SDG), a phytoestrogenic lignan. It has antioxidant properties that can protect the body from free radicals’ production and act as an anticarcinogenic agent [4]. This lignan is converted by the gut microbiota into enterolignans, which show the characteristic properties of mammalian lignans enterodiol (END) and enterolactone (ENL). Flaxseed lignans possess both estrogenic and antiestrogenic properties, along with antioxidant effects [19,20], which contribute to their potential to inhibit the growth of cancerous tumors. Their impact is particularly notable in hormone-sensitive cancers, such as those affecting the breast, endometrium, and prostate [20]. Additionally, flaxseed lignans, particularly SDG, have shown promise as an adjuvant in the prevention and management of diabetes. Studies conducted in vitro and on animal models have demonstrated their potential benefits. Wang et al. investigated the effects of SDG on glucose homeostasis in obese mice and found that it significantly reduced fasting blood glucose, insulin, and free fatty acid levels while enhancing oral glucose tolerance and insulin responsiveness [21].

It also can be responsible for gene expression modulation, impacting enzyme activity. Not only can SDG influence gene expression and the activity of genes involved in metabolic processes related to diabetes, but it also exerts effects on both normal and cancerous tissues. This offers an additional mechanism beyond its antioxidant properties for combating cancer. SDG also showed potential anti-inflammatory properties in vitro on lipoxygenase (LOX) and human cyclooxygenase (COX-2) enzyme inhibition, known as pro-inflammatory enzymes [19].

SDG, a recognized phytoestrogen found in flaxseed, has been reported to exhibit mild antihypertensive effects. It functions as a long-acting hypotensive agent by activating guanylate cyclase pathways, which are essential for regulating blood pressure [22].

#### 3.2.2. Phenolic Acids and Flavonoids

Phenolic compounds are commonly found in plants and are frequently associated with numerous health benefits, primarily due to their strong antioxidant effects. Phenolic acids and flavonoids demonstrate a wide range of therapeutic effects, including antimicrobial, anti-inflammatory, antithrombotic, antiallergenic, anti-atherogenic, antioxidant, cardioprotective, and vasodilatory properties [23]. These antioxidant components also prevent the photo-oxidation in whole and ground flaxseed, preserving these seeds for longer times [4].

The values of phenolic acids depend on the cultivar and environment. Flaxseed phenolic acids primarily consist of p-hydroxybenzoic acid (1719 mg/100 g), with substantial contributions from chlorogenic acid (720 mg/100 g), ferulic acid (161 mg/100 g), and coumaric acid (87 mg/100 g) [23].

Flavonoids include various subgroups such as flavonols, flavones, flavanones, isoflavones, anthocyanins, and flavanols. Their content in flaxseed also depends on the cultivar and growing conditions [4]. The highest reported content of flavonoids in flaxseed was around 71 mg/100 g and the lowest was around 35 mg/100 g [24]. The bioactivity of flavonoids depends on gut bacteria, such as *Lactobacilli* and *Bifidobacteria*, which metabolize them into bioactive compounds like herbacitin, quercetin, quercetagetin, kaempferol, naringenin, and eridictyol, which are antioxidants and may have a positive impact on health [4].

Even in minor amounts, the phenolic compounds and flavonoids found in flaxseed play a crucial role in promoting antioxidant activity, regulating blood pressure, and protecting against chronic diseases. This makes flaxseed a valuable addition to any balanced diet aimed at supporting overall well-being.

## 4. Benefits of Its Use in Diet

**Figure 2:**
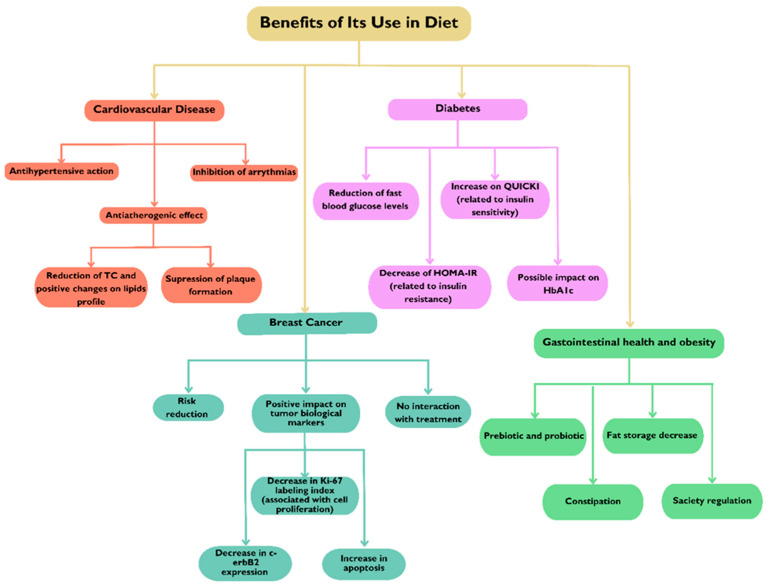
Diverse health benefits associated with incorporating flaxseed into the diet. c-erB2: human epidermal growth factor receptor 2; HbA1c: glycated hemoglobin; HOMA-IR: homeostasis model assessment-estimated insulin resistance; Ki-67: antigen kiel 67; QUICKI: quantitative insulin sensitivity check index; TC: total cholesterol.

Flaxseed offers numerous health benefits when included in a diet (Figure 2). Rich in omega-3 fatty acids, particularly ALA, it supports heart health by reducing inflammation and improving cholesterol levels. Its high fiber content aids digestion and promotes a healthy gut, while the lignans present in flaxseed provide antioxidant and hormone-balancing effects. Some specific components of flaxseed, such as ALA, lignans, and fiber, present a significant effect on the prevention and management of chronic conditions like cardiovascular disease and cancer. Research has shown that ALA, an omega-3 fatty acid, can help reduce blood pressure, lower cholesterol levels, and improve overall heart health, while lignans possess antioxidant and anti-inflammatory properties that may play a role in cancer prevention, particularly breast and prostate cancers [25,26,27]. Additionally, the high fiber content in flaxseed supports digestive health and helps regulate blood sugar levels, further contributing to cardiovascular well-being. Data from clinical studies highlight that regular flaxseed consumption may reduce the risk of these diseases, making it a valuable dietary addition for promoting long-term health. Additionally, flaxseed’s antioxidant properties help protect against chronic diseases, making it a valuable addition to a balanced diet. In the following subsections, these benefits are explored.

### 4.1. Cardiovascular Disease

Cardiovascular diseases continue to be the leading cause of death and illness globally. Functional foods are gaining increasing recognition as key components of lifestyle changes in the fight against various health conditions. These foods offer proven health benefits beyond their basic nutritional value, as they contain nutraceuticals–nutrients and bioactive compounds that contribute to these positive effects [25].

Multiple preclinical and clinical studies have demonstrated significant benefits of dietary flaxseed supplementation in this context [26]. Its consumption acts in the different aspects of cardiovascular disease, which intensifies its action against it, as flaxseed has an antihypertensive action, antiatherogenic effects, an anti-inflammatory action, and an inhibition of arrhythmias [26].

ALA is the bioactive responsible for the antihypertensive effects of flaxseed. The mechanism behind this action involves the effect of ALA on the concentration of oxylipins [27]. Oxylipins are oxygenated metabolites of PUFAs and have been implicated in the pathogenesis of hypertension since they take part in controlling inflammation and vascular tone when they are derived from omega-6 fatty acids. When oxylipins are derived from omega-3 PUFAs, they tend to have the opposite effects reducing vasoconstriction and inflammation. PUFAs can be metabolized by cyclooxygenase, lipoxygenase, and CYP450. This last one catalyzes the formation of an endothelium-derived hyperpolarizing factor associated with vasodilation through the activation of nitric oxide (NO) synthase. By enhancing vasodilation, this factor promotes a drop in blood pressure. The ALA content of these seeds can also compete with the same enzymes and receptors as omega-6 fatty acids, inhibiting its hypertensive effects [27].

While not the primary component responsible for flaxseed’s antihypertensive effects, SDG plays a significant role due to its antioxidant properties. It can mitigate the pathogenic impact of reactive oxygen species, which are involved in the development of hypertension. SDG also has other proposed mechanisms to be able to reduce blood pressure, such as the activation of guanylate cyclase and angiotensin I inhibition [26].

Where flaxseed peptides are concerned, they may be capable of inhibiting the angiotensin-converting enzyme and renin. They are also rich in arginine, an amino acid converted in vascular endothelium to nitric oxide and citrulline, causing vasodilation and contributing to flaxseed hypotensive action [26].

The FLAX-PAD (FLAX Effects in Peripheral Arterial Disease) trial was a randomized, double-blind, placebo-controlled study conducted over one year to assess the impact of daily flaxseed supplementation (30 g) on 110 patients with Peripheral Arterial Disease (PAD). Participants, all over 40 years old with an ankle–brachial index of less than 0.9, were observed for more than six months. The trial found a significant reduction in blood pressure among those receiving flaxseed, with a decrease of 10 mmHg in systolic blood pressure (SBP)—a 6.5% reduction—and 7 mmHg in diastolic blood pressure (DBP)—a 9.8% reduction. Notably, a subgroup of patients who started with elevated blood pressure (≥140 mmHg) experienced even greater reductions, with a 15 mmHg decrease in SBP and a 7 mmHg decrease in DBP, both after six months [28].

Consuming flaxseed can positively influence the serum lipid profile in humans, offering significant benefits, particularly for individuals at risk of cardiovascular disease or those already affected by it. With whole flaxseed, the majority of human studies reported reductions in TC (total cholesterol) by 6–11% and low-density lipoprotein cholesterol (LDL-c) by 9–18% in normolipidemic subjects, and TC by 5–17% and LDL-c by 4–10% in hypercholesterolemic subjects. The levels of atherogenic lipoproteins and apoproteins A-1 and B after 12 weeks of dietary intervention, 38–40 g/day in food, can be reduced. However, the FLAX-PAD trial also evaluated the effect of daily 30 g of flaxseed in foods in plasma lipids (measured at 0, 1, 6, and 12 months). A reduction of 15% and 11% in LDL-c and TC, respectively, was observed at 1 and 6 months (differences within the treatment group). At 12 months, the differences were much less significant. In a subgroup of patients taking cholesterol-lowering medication, LDL-c decreased by 8.5 ± 3.0% at 12 months. This trial also concluded that the LDL-lowering effects of flaxseed were additive to medication and a healthy lifestyle [29].

In addition, both animal studies and a meta-analysis of 28 clinical trials concluded that, unfortunately, flaxseed does not produce any significant changes in HDL-c and triglyceride levels [30]. Animal studies were also conducted to determine if flaxseed has any effects on atherosclerosis. Dupasquier et al. reported that flaxseed supplementation led to a reduction in atherosclerosis and suppression of atherosclerotic plaque formation, with these effects being dose-dependent in the diet [31]. It is also crucial to determine whether established atherosclerotic plaques can regress during a 14-week period of dietary flaxseed supplementation following plaque stabilization, resulting in, approximately, a 40% reduction in the affected area [32]. Furthermore, in another study by Dupasquier et al., a clinical setting characterized by increased vascular reactivity, the flaxseed-enriched diet significantly improved both vascular contraction and endothelium-dependent vessel relaxation [33].

Of all the main components of flaxseed, flax oil rich in ALA indicates no consistent beneficial effect on lipid-lowering. While flax fiber has been suggested to potentially increase fat excretion in feces and thereby help lower lipid levels, the effects have not been consistently significant. In contrast, SDG and other flax lignans, which are bioactive compounds, have demonstrated notable effects on lipid levels, with significant results observed in both animal and human studies. The first one demonstrated the reduction in hepatic cholesterol in a dose-dependent manner in hypercholesterolemia and the slowing of aortic atherosclerosis and atherosclerotic plaque progression [26]. Moreover, in an 8-week, randomized, double-blind, placebo-controlled study conducted in 55 patients, all hypercholesterolemic, previously divided into three groups of daily supplementation of SDG from flaxseed of 0 mg (placebo), 300 mg, and 600 mg, this compound demonstrated a possible beneficial effect on plasma lipids. At weeks 6 and 8, the 600 mg SDG group showed a reduction of approximately 22% in total cholesterol (TC) and 24.38% in LDL cholesterol (LDL-c) compared to the placebo group. In the 300 mg SDG group, significant reductions in TC and LDL-c were only observed when compared to baseline levels. It was concluded that cholesterol levels relate to dietary flaxseed SDG in a dose-dependent manner [34].

The lignan content also had a beneficial effect on the lipid profile of participants. A 6-week, randomized, double-blind, placebo-controlled study examined the impact of lignans on cardiovascular risk factors. A total of 37 participants (13 men and 24 women, average age of 54 ± 7 years, BMI of 29.7 ± 1 kg/m^2^) consumed nutrition bars with similar macronutrient profiles (including 3.0 g of ALA) but different lignan contents, 0.15 g versus 0.41 g. The high-lignan flaxseed bars resulted in significant reductions of 12% in total cholesterol (TC), 15% in LDL cholesterol (LDL-c), and 25% in oxidized LDL (Ox-LDL). In contrast, the regular flaxseed bars showed a tendency to increase Ox-LDL levels. The difference between the high-lignan and regular-lignan bars on Ox-LDL was statistically significant, which is particularly important because Ox-LDL is an independent cardiovascular risk factor, considered more atherogenic than LDL-c itself [35].

The phytosterols activate another lipid-lowering mechanism of flaxseed, present in the amount of 338 mg for every 100 g of flaxseed; these bioactive compounds are structurally similar to cholesterol, which makes them compete with it to be absorbed in the intestinal tract, and this leads to an increase in the fecal excretion of cholesterol and a reduction in its levels [25].

Flaxseed fiber has a lipid-lowering effect because its consumption creates satiety; therefore, the caloric intake is reduced and so is the lipid one. Fiber also reduces transit time, increases bile acid excretion, and decreases bile acid reabsorption via increased fecal cholesterol excretion [26].

Antiplatelet effects would also be a beneficial outcome of the consumption of flaxseed; however, there is a lack of studies on this matter both animals and humans. Flaxseed oil rich in ALA has demonstrated a significant reduction in platelet aggregation, including inhibition of thrombin- and collagen-induced platelet aggregation, which is the case of ALA in animal studies [36]. In contrast, human studies have shown no effects on platelets or isolated cases of significant inhibition of aggregation or even significant effect, but in small sample sizes; therefore, it is important to study the impact of flaxseed in this area and its mechanisms [26].

In the reported animal studies, a whole flaxseed-supplemented diet resulted in a lower incidence of arrhythmias and a shorter QT interval after the heart was subjected to ischemia and reperfusion [37]. Flax oil rich in ALA revealed a cardioprotective effect in ischemia and reperfusion. ALA decreased infarct size, apoptosis, and TNF-α and IL-6 concentrations, and increased the anti-inflammatory cytokine IL-10 and cardiac antioxidant enzymes, and, consequently, inflammation and apoptosis [38]. ALA may also be the bioactive responsible for the antiarrhythmic effects of flaxseed because of omega-3 PUFA’s action on the phospholipid bilayer of the myocardial cells and its role in depolarization [39]. SDG was shown to significantly enhance the expression of vascular endothelial growth factor (VEGF), angiopoietin-1, and phosphorylated endothelial nitric oxide synthase in the myocardium. VEGF plays a crucial role in stimulating blood vessel formation and may help improve endothelial cell survival. Endothelial nitric oxide synthase is thought to mediate VEGF’s effects. Additionally, angiopoietin-1 promotes endothelial cell survival by reducing apoptosis. These factors collectively contribute to the restoration of vascular responsiveness in ischemic tissue, leading to improved myocardial perfusion and cardiac function [26].

### 4.2. Diabetes

Although there are two types of diabetes mellitus, the effects of flaxseed were mainly evaluated in diabetes mellitus type 2 (DMT2). This endocrine metabolic disorder affects the metabolism of carbohydrates, primarily glucose, but also lipids and proteins. Glucose metabolism is affected by insulin secretion and action, which in DMT2 are compromised. The main characteristic of all types of diabetes is hyperglycemia or fast blood glucose with values above 126 mg/dl. Long-term hyperglycemia leads to macrovascular and microvascular complications such as diabetic retinopathy, nephropathy, neuropathy, or atherosclerosis, which are great causes of morbidity and mortality worldwide. So, to complement pharmacological therapy, there is a growing interest in what role functional foods, like flaxseed, can play in these types of diseases [40].

Many randomized clinical trials have demonstrated the benefits of flaxseed on glycemic control and insulin sensitivity, even though more data are needed, and the research has been increasing because of the potential that flaxseed has revealed in improving glycemic levels.

The aforementioned 8-week, randomized, double-blind, placebo-controlled study involved 55 hypercholesterolemic patients who were divided into three groups receiving daily supplementation of SDG from flaxseed at doses of 0 mg (placebo), 300 mg, or 600 mg. The study also evaluated the impact of SDG on fasting glucose levels. Significant reductions of 25.56% and 24.96% were observed at weeks 6 and 8, respectively, compared to both placebo and baseline. These decreases were greater in individuals with baseline glucose levels of 105 mg/dL. In the group with the 300 mg daily supplementation, the lowering effect of fasting glucose levels was noticeable, but not statistically significant. As the plasma cholesterol, the decrease in glucose concentration is related to dietary flaxseed SDG in a dose-dependent manner [34]. An updated systematic review and meta-analysis of 53 randomized clinical trials revealed significant effects on reducing fasting blood glucose, HOMA-IR (homeostasis model assessment of insulin resistance), and serum insulin levels, while increasing QUICKI (quantitative insulin sensitivity check index). These effects were notably more pronounced when compared to control groups or placebo, particularly within subgroups [40]. However, a significant reduction in HbA1c was not found, which could be due to the short duration of some studies, since HbA1c is the index for evaluating glycemic response in the last three months. Nevertheless, in a subgroup analysis where studies had a duration superior to 12 weeks, whole flaxseed significantly reduced these levels compared to placebo or control. Despite the good results, in all the subgroups’ analyses, heterogeneity was high, which does not permit an accurate interpretation, as wished, of these results. It is important to note that most of these studies were conducted in patients whose pathogenesis of the disease was related to insulin resistance, so the results have shown progress in reducing insulin resistance and improving insulin sensitivity.

Various mechanisms were mentioned to explain how flaxseed produces its effect on glycemic indices, these being the α-glucosidase and α-amylase inhibition (studies in vivo and in vitro), and flaxseed polyphenols helped in the recovery of pancreas, liver, pancreatic β-cells, and kidney functions (in vivo study in diabetic rats and histopathological investigations). Whole flaxseed has a great percentage of fiber, which delays glucose absorption and improves hormone response. Fibers can also produce short-chain fatty acids and regulate gut microbiota, positively affecting glycemic levels [40]. Flaxseed oil, rich in omega-3 fatty acids, has been shown to modulate anti-inflammatory responses and influence gut microbiota in vivo [41]. Additionally, polyunsaturated fatty acids (PUFAs) act as natural agonists of proliferator-activated receptor-γ (PPARγ), which plays a crucial role in glucose metabolism and insulin sensitivity. PUFAs also activate G-protein-coupled receptors (GPRs), which stimulate the increased secretion of glucagon-like peptide-1 (GLP-1) [40].

SDG may have an important role, too, as its hypoglycemic effect is played via the inhibition of PEPCK (phosphoenolpyruvate carboxykinase) gene expression; this way, PEPCK, an enzyme involved in the glucose production by the liver (glucogenesis), is not coded and there is less production of glucose, which decreases glycemic levels [19].

If some systematic reviews and meta-analyses show no effects of flaxseed in HbA1c, others affirm the opposite. Of 13 studies included, the results indicate a decrease in HbA1c in participants with a baseline of more or equal to 7.0% after flaxseed supplementation, especially with poorly controlled DMT2, which may mean that this supplementation is an ally in the control of the disease, even in worsen periods [42].

Effective diet management is widely recognized as the cornerstone of treating type 2 diabetes mellitus (DMT2) and its associated complications. Although further randomized clinical trials and consistent results are necessary to fully understand the potential of flaxseed supplementation on DMT2 and related cardiometabolic parameters, existing data already provide valuable insights. These findings offer promising recommendations that could be beneficial for complementing the diets of diabetic patients [42].

### 4.3. Breast Cancer

Cancer poses one of the most significant public health challenges due to its high and rising prevalence. As a leading cause of morbidity and mortality globally, it not only significantly impacts life expectancy but also greatly diminishes quality of life. Nutrition is fundamental during cancer treatment and can prevent complications and hopefully reduce the severity of side effects, improving the patients’ well-being.

In this context, flaxseed has been one of the most studied foods regarding its role in breast cancer, one of the most common cancers, and the second with the biggest mortality rate worldwide. This happens because the seeds are characterized by their lignans content, 95% of which is SDG, and may have a protective effect against breast cancer due to their low estrogenic activity and antioxidant properties [43]. The former property is related to enterolactone and enterodiol, mammalian lignans that result from the conversion of SDG in the colon by bacteria [19]. There are studies that demonstrate a significant association between serum enterolactone levels and the risk reduction in breast cancer [43]. These metabolites share structural similarities to the main form of estrogen, which allows their binding to the cell receptors, displacing estrogen from cells. Therefore, these lignans work as antiestrogens, preventing estrogen from binding to the receptors and inhibiting the growth of cancer cells. Estrone, the biologically active form of estrogen, is converted into two different metabolites: 2-hydroxyestrone (2OHE1) (with small biological activity) and 16α-hydroxyestrone (16OHE1), which enhances estrogen activity and promotes cell proliferation, particularly in estrogen receptor-positive (ER+) breast tumors. The displacement of estrogen by lignans, such as those from flaxseed, can help reduce the production of 16OHE1, potentially decreasing the risk or severity of estrogen-dependent cancers [43].

A randomized crossover trial involving 28 postmenopausal women, aged 53 to 82 years, was conducted to assess the effects of ground flaxseed supplementation. The study comprised three 7-week feeding periods: a control period where participants followed their usual diet, and two experimental periods where the usual diet was supplemented with either 5 g or 10 g of ground flaxseed. Each feeding period was separated by a washout period of at least 7 weeks. Despite the small biological activity of 2OHE1, it was suggested that this metabolite is protective against breast cancer, suppressing the growth and proliferation of breast cancer cells. So, the ratio of 2/16-OHE1 was used as a biomarker for breast cancer risk, with an increase in its value considered protective. And 2OHE1 excretion showed a linear, dose–response increase between the control, the 5 g, and 10 g feeding periods, and significantly higher for the 10 g feeding period. The same was observed for the 2/16-OHE1 ratio. The 16OHE1 excretion was not significantly affected. This study concluded that flaxseed supplementation may be protective against breast cancer [44].

A randomized, double-blind, placebo-controlled clinical trial was conducted to examine the effects of dietary flaxseed on tumor biological markers and urinary lignan excretion in postmenopausal women newly diagnosed with breast cancer. The treatment group (n = 19) consumed a daily muffin containing 25 g of flaxseed, while the control group (n = 13) consumed a placebo muffin. Tumor tissue was analyzed at the time of diagnosis and again at the time of definitive surgery for key markers, including the tumor cell proliferation rate (Ki-67 labeling index, the primary endpoint), apoptosis, c-erbB2 expression, and estrogen and progesterone receptor levels. Additionally, 24 h urine samples were collected and analyzed for lignan levels. The average treatment duration was 39 days for the placebo group and 32 days for the flaxseed group. Results showed a significant reduction in the Ki-67 labeling index and c-erbB2 expression, along with an increase in apoptosis in the flaxseed group. Furthermore, urinary lignan excretion was notably higher in this group. The study concluded that dietary flaxseed may have the potential to reduce tumor growth [45].

Moreover, ingestion of ALA, of which flaxseed is one of the best sources, has been associated in studies with the reduction in the risk of breast cancer, and animal studies demonstrated suppression of growth, size, cell proliferation, and an increase in its death [43].

It is also crucial to explore whether flaxseed interacts with drugs used in breast cancer treatment, such as tamoxifen. While there are no clinical trials available, experimental studies have not demonstrated any adverse interactions, and flaxseed may offer protective effects. In animal studies, flaxseed kept or increased tamoxifen action in decreasing tumor growth, cell proliferation, and increased apoptosis [43]. A pilot study including postmenopausal women with ER+ breast cancer was also conducted to evaluate the effects of flaxseed in the treatment with anastrozole, and, although further studies are still needed, no interaction was noticed [46].

At first sight, based on existing clinical trials that examine the relationship between flaxseed intake and its effects on breast cancer, the results are encouraging to include flaxseed in the diet due to its apparent beneficial role, owing to its lignan composition. However, these phytoestrogens, apart from their anti-estrogenic activity, can also have estrogenic properties, which raises concerns among healthcare professionals and scientists since further studies are still needed and the current evidence is mixed. That being said, the safety of phytoestrogens in people with a history of hormone-positive cancers is not well established and it is not possible to predict how each individual organism with variable characteristics is going to react, so it is important to consult a healthcare provider before incorporating flaxseed into the diet to balance potential benefits and risks.

### 4.4. Gastrointestinal Health and Obesity

Gastrointestinal health plays a crucial role in maintaining human health, immunity, cognitive function, and mental well-being, as its microbiota is involved in the production of various substances, including neurotransmitters, specific metabolites, brain-derived neurotrophic factors, short-chain fatty acids, tryptophan, and gamma-aminobutyric acid (GABA) [47]. These substances are essential for nervous system coordination, as the brain, nervous, digestive, and endocrine systems are interconnected through sympathetic and parasympathetic nerves, forming the brain–gut axis. This axis directly influences the physiological functioning of bodily organs and the immune system. Therefore, maintaining proper regulation of the gut can help prevent stress-related disorders, endocrine imbalances, and inflammatory diseases, and improve the prognosis of various health conditions [47].

To guarantee gastrointestinal health, guaranteeing the preservation of the gut microbiome or its enhancement is crucial. One of the most significant genera and a major component of the gut microbiota is *Bifidobacteria*. These beneficial bacteria play a crucial role in the digestion and assimilation of carbohydrates and lipids, contributing to overall metabolic health [47]. Other generas are *Bacillus*, *Clostridium*, *Klebsiella*, *Eubacterium*, *Peptostreptococccus*, *Ruminococcus*, *Nocardia*, and *Streptomyces*.

The consumption of flaxseed can positively impact the gut microbiome since this functional food can work as a prebiotic. Prebiotics are nutrients that feed the intestinal microbiota and give it the substrate to support its activity. The maintenance of the gut microbiome activity and thus regulating and stabilizing these microorganisms can bring benefits to human health. When consumed, flaxseed polysaccharides can gradually alter the composition of the intestinal microbiota, promoting the growth of bacteria that metabolize these compounds, such as Akkermansia, Bifidobacterium, Clostridium, Enterococcus, Lactobacillus, Megamonas, Phascolarctobacterium, and Prevotella species. This shift has been shown to play a significant role in reducing inflammation and colitis, repairing the gut lining, enhancing insulin sensitivity, protecting against intestinal tumors, and slowing the progression of disease [47].

SDG, as mentioned above, is also transformed into enterodiol and enterolactone by the action of various bacteria (*Prevotella*, *Akkermansia*, and *Bifidobacterium*), compounds that have a significant role in human defense mechanisms against disease [47].

It was also found that flaxseed promotes the increase in the percentage of omega-3 fatty acids in the diet, which leads to an abundance of *Bifidobacteria* in the colon. Gut bacteria synthesize fatty acids that will be used in different processes or become part of triacylglycerols (TAG), energy-storage molecules present in the cell cytoplasm [47]. However, ALA, an essential amino acid that composes TAG and is a building block of other long-chain polyunsaturated fatty acids, cannot be synthesized in the human body, and it has to be acquired from external sources, like flaxseed. The gut microbiota also participates in the metabolism of PUFAs and ALA.

Flaxseed also plays a role in obesity prevention. This functional food can positively influence the gut microbiota of individuals. A key factor is the balance between Firmicutes and Bacteroides in the intestines, with an increased ratio of Firmicutes to Bacteroides linked to greater energy extraction from food and higher triglyceride storage in tissues [48]. Flaxseed fiber and mucilage help reduce this ratio, promoting a healthier balance of bacteria. Additionally, flaxseed helps regulate blood sugar levels, decrease fat storage, and enhance satiety, all of which contribute to weight loss and obesity prevention [47].

However, further in vitro, in vivo, and nutritional intervention studies are necessary to better understand the effects of flaxseed on the gut microbiome and its potential to improve human health and prevent various diseases [47].

Constipation has become increasingly common, particularly among middle-aged and elderly populations and women. A clinical trial investigating the impact of flaxseeds on the gut microbiota in 60 elderly patients (54 men and 6 women, average age of 68.68 ± 8.73 years) with functional constipation showed that consuming 50 g of flaxseed daily for one month significantly improved defecation frequency, reduced abdominal distension, improved stool consistency, and decreased the need for laxatives compared to pre-treatment symptoms in patients with chronic constipation [49].

Fibers are well known for improving gut motility and reducing defecation time. However, flaxseed goes further by significantly enhancing the diversity of bacterial clusters in the gut. Specifically, it increases beneficial probiotics like *Akkermansia* and *Bifidobacterium* while decreasing harmful bacteria such as *Clostridium perfringens* [49]. This shift, along with the elevated content of short-chain fatty acids, can help reduce the risk of developing conditions such as colitis and colon cancer [49]. These findings indicate that flaxseed might regulate gut microbiota as a probiotic and showed no adverse reaction during the period of treatment, and its therapeutic efficacy was better than that of lactulose [49].

Diet intervention plays a crucial role in constipation management and gastrointestinal health, and flaxseed stands out as an optimal supplement because of its safety, effectiveness, and convenience.

## 5. Potential Toxicity

While the consumption of flaxseed offers significant health benefits, it is equally important to be aware of the potential risks associated with its chronic use and its interactions with medicines and other mechanisms in the human body.

The toxicity of flaxseed can come from overconsumption of its valuable compounds, interactions, toxic factors, or possible contaminants. Consulting a nutritionist, doctor, or pharmacist about incorporating flaxseed into one’s regular diet can be beneficial for everyone, regardless of their medical history or current medication use. This personalized guidance ensures a thorough evaluation of potential health benefits.

The main counter-indications of flaxseed meal include lactating and pregnant women because of its activity as estrogens, responsible for inducing labor and low birth weight. Due to these effects, flaxseed can also interfere with birth control pills or hormone replacement therapy. In the case of ALA, which promotes blood flow, it can have a synergistic effect with blood-thinning medicines and increase the risk of bleeding. When consumed alongside antidiabetic medications or insulin, flaxseed may amplify the hypoglycemic effect more than anticipated, potentially leading to hypoglycemia and its associated risks, which can be both unwanted and hazardous [4].

In an experimental study, several participants decided to withdraw due to unpleasant gastrointestinal effects that occurred within 4 weeks caused by whole flaxseed and oil preparations. This highlights the potential for excessive flaxseed intake to cause gastrointestinal disturbances and underscores the varying effects of different forms of flaxseed. In fact, milled flaxseed was associated with fewer adverse effects compared to whole flaxseed and oil [50].

Apart from that, flaxseed also holds in its composition antinutritional compounds, mainly, cyanogenic glycosides transformed in hydrogen cyanide, phytic acid, and trypsin inhibitors.

### 5.1. Phytic Acid

Phytic acid is the main storage form of phosphorus not only in oil seeds, but also in cereals, legumes, and nuts, comprising 50–80% of the total phosphorus in these plants. Its main adverse effect is interfering with the absorption of essential minerals such as calcium, zinc, magnesium, copper, and iron, as well as proteins. This interference occurs because phytic acid chelates these minerals and, consequently, reduces their bioavailability to monogastric animals, including humans, who lack the enzyme phytase necessary to break down phytic acid [51].

Nissar et al. reported that phytic acid should be lower as much as possible and recommended consuming 25 mg or less of this antinutrient per 100 g of food. However, the recommended daily intake of phytic acid varies from country to country; for example, the United Kingdom and the United States present values that range between 631 and 746 mg/day, while, on the other side, Sweden recommends only 180 mg/day [52].

According to Khare et al., phytic acid was present in flaxseed in the range of 770–920 mg/100 g. In this study, four varieties of flaxseeds were analyzed [53].

### 5.2. Trypsin Inhibitors

Trypsin inhibitors are natural metabolites that protect flaxseed from biological stress. These compounds inhibit the activity of key pancreatic enzymes, trypsin, and chymotrypsin, resulting in a decrease in protein digestion and absorption by forming indigestible complexes [54]. The reduction in crucial proteins involved in growth in maintenance may lead to poor and diminished growth of humans and animals. In more extreme cases, to compensate for protein reduction, there is an overstimulation of the pancreas to produce more enzymes, which may cause pancreatic hypertrophy [55]. For decades, the presence of these antinutritional compounds has been studied, since their control should be a health concern.

The study by Khare et al. also determined the amount of trypsin inhibitors present in flaxseed, ranging from 22.78 to 28.85 mg/100 g [53].

To ensure the safety and health benefits of flaxseed, it is crucial to advance detection, quantification, and detoxification techniques. Despite the significant potential and numerous health advantages of flaxseed, its content of various antinutritional compounds requires careful consideration. This monograph will give special attention to the presence of cyanogenic glycosides; therefore, a special section will be dedicated to these contaminants.

## 6. Cyanogenic Glycosides

Cyanogenic glycosides are nitrogenous secondary plant metabolites that include linamarin, linustatin, lotaustralin, and neolinustatin [56]. These compounds consist of an α-hydroxynitrile aglycon and a sugar moiety (glucose or gentiobiose), which accumulate in vacuoles and, upon cell destruction by ingestion, the glycone portion is removed by hydrolysis performed by intestinal β-glucosidase [26]. The obtained cyanohydrin is degraded to hydrogen cyanide (HCN). This newly produced compound can be toxic to the respiratory, nervous, and endocrine systems, as HCN binds to iron, manganese, or copper ions in various enzymes, including those critical to the cytochrome respiratory chain [26,57]. Apart from being a respiratory inhibitor, HCN can also be converted into thiocyanates that interfere with iodine uptake by the thyroid gland, and long-term exposures may lead to iodine-deficiency disorders such as goiter or cretinism [58].

These compounds serve as a natural defense mechanism for flaxseed, and despite the calculations indicating minimal risk, it is essential to ensure that the remaining cyanogenic glycosides pose no threat to human health. This can be achieved through rigorous detection, quantification, and detoxification techniques.

### 6.1. Determination Techniques

Nine articles from 1996 to 2023 were analyzed and are detailed in Table 1 and Table 2.

The extraction techniques used for cyanogenic glycosides (CNGs) in flaxseed primarily included solid–liquid extraction, solid-phase extraction (SPE), distillation, and derivatization to prepare trimethylsilyl (TMS) derivatives for further analysis. Except for distillation (which lacks available data), all these methods demonstrated good recovery values.

The determination and quantification methods for CNGs can be divided into two categories: direct and indirect methods. Direct methods allow for the differentiation and quantification of cyanogenic glycosides typically present in flaxseed (linustatin, neolinustatin, linamarin, and lotaustralin), as well as others (amygdalin, prunasin, epilotaustralin). These methods mainly involve liquid chromatography (LC), specifically high-performance liquid chromatography (HPLC) and ultra-high-performance liquid chromatography (UHPLC), often coupled with mass spectrometry (MS), often with electrospray ionization (ESI-MS). Quantitative nuclear magnetic resonance (qNMR) and gas chromatography (GC) have also been employed for these purposes. The advantages of liquid chromatography (LC) over gas chromatography (GC) for the determination of cyanogenic glycosides include LC’s ability to analyze non-volatile, thermally labile compounds without the need for derivatization, offering higher sensitivity and specificity for these types of compounds.

Indirect techniques, such as ion chromatography and colorimetric methods, measure the hydrogen cyanide (HCN) content that results from the degradation of cyanogenic glycosides. These methods do not directly quantify the cyanogenic glycosides themselves but, rather, assess the HCN produced from their breakdown.

Evaluating the values of limit of detection (LOD), limit of quantification (LOQ), and relative standard deviation (RSD), alongside the accuracy (evaluated through recovery assays) of the corresponding extraction methods, liquid chromatography techniques stand out as the most sensitive, accurate, precise, and reliable. These techniques also exhibit good repeatability and have been validated and successfully applied not only to flaxseed but also to its products and other foods.

Between the methods that identified the major number of different cyanogenic compounds, one qNMR and a UHPLC-QqQ-MS/MS, the UHPLC-QqQ-MS/MS from Zhong et al. outperformed in terms of sensitivity, achieving better limits in terms of LODs (1–25 ng/g) and LOQs (5–100 ng/g), and demonstrating good recovery rates (89.88–125.69%) [59]. When applied to flaxseed samples, not all eight cyanogenic glycosides were detected. Only four compounds, linamarin, lotaustralin, linustatin, and neolinustatin, were found in all samples. Among these, linustatin (0.22–2.83 mg/g) and neolinustatin (0.29–3.65 mg/g) were identified as the main cyanogenic compounds [59]. The UHPLC-QqQ-MS/MS method provides the first validated method (as of the publication date) for direct and simultaneous quantification of these eight cyanogenic glycosides in cyanic agri-foods. It demonstrated good sensitivity, precision, and accuracy, and was successfully applied to various cyanic agri-foods, including flaxseed [60]. However, it should be noted that qNMR is an efficient and rapid (≈20 min) tool to quantify cyanogenic glycoside content in flaxseed, which makes it possible to incorporate it into the routine [61].

The UHPLC-MS/MS method from Cai et al. is more sensitive than the UHPLC-QqQ-MS/MS method, even though it identified only four cyanogenic compounds—linamarin, lotaustralin, linustatin, and neolinustatin—in flaxseed [62]. These four compounds were the only ones found in flaxseed in the previous method, too. Cai et al. compared their UHPLC-MS/MS method to UHPLC-QqQ-MS/MS and aimed to solve the matrix effects [62]. Instead of using a traditional Prime HLB (hydrophilic–lipophilic-balanced copolymer) column, they employed a cigarette filter for purification in the solid-phase extraction (SPE) before chromatography analysis. This approach resulted in limits of detection (LODs) between 0.13 and 0.44 pg/g and limits of quantification (LOQs) between 0.44 and 1.48 pg/g, indicating very high sensitivity. Additionally, the method achieved good recovery rates (113–133%) [62]. The UHPLC-MS/MS method was validated and found to be simple, easy to perform, accurate, reliable, highly sensitive, and stable, with broad applicability [62].

**Table 1:** Compilation of methods to determine cyanogenic compounds in flaxseed or flaxseed products.

Samples	Cyanogenic Compounds	Extraction	Analytical Technique	LOD	LOQ	Recovery	Precision (RSD)	Year	Ref.
Flaxseed from a previous study, ethanol-extracted ground flax, flax pressed cake, spent flaxseed, and CO_2_-extracted flaxseed	Linamarin, linustatin, and neolinustatin	See Table 2.	HPLC-RI Analytical column: RP-18 (4 × 250 mm, 10 µm particle size). Mobile phase: 95/4.95/0.05 H_2_O/methanol/H_3_PO_4_. Injection volume: 20 µL. Flow rate: 0.7 mL/min. Temperature: 21 °C. Isocratic mode.	N.A.	N.A.	N.A.	37.41%	1996	[63,64]
Mature oil-type flaxseed from 7 different cultivars grown in 2 locations in 3 different years	Linustatin	Extraction solvent: methanol/H_2_O 3:1 (*v*/*v*). Four successive extractions with a ratio of 6:1 of solvent volume to the mass of ground seed, with an ultrasonic bath for 30 min at 40 °C. Centrifugation between each extraction: 10 min, 4 °C, 3824× *g*.	qNMR Dried samples + 1.5 mL methanol/D_2_O 3:1 (*v*/*v*) + analysis standard: TMSP 0.1 mg/mL. Homogenization and centrifugation at 20,000× *g* and 4 °C for 10 min. Supernatant was collected in a 5 mm NMR tube. _1_H and _13_C assignments for linustatin, neolinustatin, and related compounds.	149.4 μg/g	249.1 μg/g	95.41 ± 1.93%	<6%	2017	[61]
	Neolinustatin			230.8 μg/g	384.6 μg/g				
	Linamarin			7.1 μg/g	11.8 μg/g				
	Lotaustralin			7.6 μg/g	12.7 μg/g				
	Epilotaustralin			6.3 μg/g	10.6 μg/g				
	Amygdalin			193.8 μg/g	323.0 μg/g				
Linforce^®^ (flaxseed coated with two herbal extracts—*Senna alexandrina mill* and *Frangula alnus*) and flaxseed	Linustatin	UAE: ultrasonic bath with 160 W; sonication cycle: 30 s ON, 10 s OFF at room temperature. Centrifugation: with conical rotor at 7000 rpm at room temperature. Cryogrounding: with a freezer/mill; duration time: 9 min (3 × 3 min); frequency: 10 impacts/s; stored at −20 °C until use. Three-step preparation method: 1. Aqueous methanol UAE (methanol/H_2_O (75/25, *v*/*v*)), resulting supernatant I (after centrifugation) and residue; 2. Residue submitted to alkaline UAE (0.08 M NaOH/methanol (25/75, *v*/*v*)), resulting supernatant II (after centrifugation); the combined extract solutions were hydrolyzed by 0.02 M NaOH, acidified (pH 2–3), diluted, and filtered.	UHPLC/ESI-HRMS Analytical column: BEH C_18_ column (2.1 × 100 mm, 1.7 um, 130 Å). ** Mobile phase A: Milli-Q water/formic acid (99.9/0.1, *v*/*v*), containing also 50 µM NaCl. Mobile phase B: acetonitrile/formic acid (99.9/0.1, *v*/*v*), containing also 50 µM NaCl. Gradient mode; flow rate: 0.2 mL/min. Temperature of the auto sampler: 6 °C. Volume of injection: 5 µL. ESI in positive mode; capillary potential: 4000 V; end-plate offset: 500 V; nebulizing gas: nitrogen, pressure at 276 K Pa; drying gas: nitrogen at 9.0 L/min; temperature: 200 °C.	2.0 µg/g	6.6 µg/g	92.3–102.5%	Repeatability precision: 1.1–6.9%. Intermediate precision: 2.4–6.6%.	2019	[60]
	Neolinustatin								
Field-grown flaxseed from 5 different cultivars and chamber-grown flaxseed from other 5 different cultivars	Linustatin	Derivatization of CNGs/preparation of TMS derivatives: Reaction: the defatted seed powder + HMDS + TMCS + IMD in a microcentrifuge tube for 15 min at 50 °C in a sonicating water bath. Centrifugation: 20,817× *g* for 5 min. Preparation for LC-MS/MS: an aliquot of the silylated extract + mobile phase was put in a microcentrifuge tube. Centrifugation: 14,000 rpm for 5 min. The diluted extract was transferred to a glass vial.	For quantification: GC–Flame ionization detector (FID) Analytical column: a capillary column (50% phenyl methylpolysiloxane, 30 m × 0.32 mm, 0.25 mm film thickness). Injection volume: 1 µL. Temperature gradient: increased from 190 °C to 280 °C at 50 °C/min. Held for 2.2 min, for an analysis of 4 min. Carrier gas: hydrogen. Flow rate: 5 mL/min. Injector temperature: 275 °C. Split ratio: 50:1. FID temperature: 300 °C. Nitrogen (make-up gas) + hydrogen + air flow rates: 30, 40, 430 mL/min, respectively. For characterization: HPLC-MS/MS Analytical column: 50 mm × 2.0 mm Fast Gradient RP-18e. Mobile phase: 100% acetonitrile. Injection volume: 3 µL. Flow rate: 0.4 mL/min. Temperature: ambient temperature. MS analysis: micrOTOF-Q II hybrid quadrupole time-of-flight MS/MS with ESI ion source operated in an MRM mode.	6.43 µg/mL	19.50 µg/mL	In 10 mg matrix: 79.9–107.4%. In 20 mg matrix: 94.2–112.7%.	<15%	2019	[65]
	Neolinustatin			4.72 µg/mL	14.31 µg/mL				
Flaxseed purchased from a supermarket	Linamarin	The samples were ground, dissolved in 80% methanol aqueous solution, placed in an ultrasonic bath, and centrifuged; this extraction was repeated twice. Solid-Phase Extraction (clean-up) Solid-phase extraction column: Prime HBL. Washing solution: 5 mL of water and 2 mL of 10% methanol aqueous solution. Elution solution: methanol/acetonitrile (30:70, *v*/*v*). The elution was repeated 3 times. Eluate was collected and evaporated at 40 °C to dryness under nitrogen. The resultant residue was redissolved in 10% methanol aqueous solution.	UHPLC-QqQ-MS/MS Analytical column: C_18_ RRHD (2.1 mm × 50 mm, 1.8 µm). *** Mobile phase A: water with 0.1% (*v*/*v*) formic acid. Mobile phase B: acetonitrile. Gradients used: isocratic and linear. Flow rate: 0.3 mL/min. Injection volume: 10 μL. Column temperature: 35 °C. MS analysis: QqQ MS equipped with ESI.	5 ng/g	20 ng/g	89.88–125.69%	Intra-day: <10.38% Inter-day: <11.33%	2020	[59]
	Lotaustralin			1 ng/g	5 ng/g				
	Linustatin			5 ng/g	20 ng/g				
	Neolinustatin			5 ng/g	20 ng/g				
	Taxiphyllin			5 ng/g	20 ng/g				
	Amygdalin			2 ng/g	10 ng/g				
	Dhurrin			2.5 ng/g	10 ng/g				
	Prunasin			25 ng/g	100 ng/g				
9 cold-pressed flaxseed oil from “Jingdong Mall”	Linustatin	Solid-Phase Extraction 1. SPE column filled with 120 g of cigarette filter fiber conditioned with 5% (*v*/*v*) isopropanol/n-hexane solution. 2. Loaded with the sample oil diluted in 5% (*v*/*v*) isopropanol/n-hexane solution. 3. Washed with 5% (*v*/*v*) isopropanol/n-hexane solution. 4. Desorption of the CNGs with methanol. 5. Blow-drying under nitrogen of the eluent, redissolution in 30% methanol aqueous solution.	UHPLC-MS/MS Analytical column: HSS T3 column (2.1 mm × 100 mm, 1.7 μm) **** Mobile phase A: water; Mobile phase B: acetonitrile. Temperature: 40 °C. Injection volume: 10 μL. Flow rate: 0.4 mL/min. Gradient mode. Detection with an ESI in the negative mode under selective detection mode (SDM). Voltage: 2800 V. Capillary temperature: 350 °C. Dry temperature: 300 °C.	0.23 pg/g	0.76 pg/g	113–133%	0.8–20.5%	2022	[62]
	Neolinustatin			0.13 pg/g	0.44 pg/g				
	Linamarin			0.43 pg/g	1.43 pg/g				
	Lotaustralin			0.44 pg/g	1.48 pg/g				
Flaxseed, flaxseed cake, and products containing these ingredients	Amygdalin	Extraction solvent: 1% formic acid in methanol/water (25/75) (*v*/*v*). Extraction: 30 min on a rotary tumbler. Centrifugation: 10 min at 3000 rpm. The supernatant is transferred to a filter vial and diluted.	UHPLC-MS/MS Analytical column: BEH C_18_ (100 × 2.1 mm, 1.7 µm). Mobile phase A: 0.1% formic acid in water. Mobile phase B: methanol (acetonitrile can also be used in small retention times). Column temp.: 50 °C. Flow rate: 0.4 mL/min. Injection volume: 2–5 µL. Gradient mode. ESI operated in positive mode; performing MRM: capillary voltage: 30 kV; cone voltage: 30 V; source temperature: 150 °C; desolvation temperature: 600 °C; cone gas flow: 150 L/h; desolvation gas flow: 800 L/h; CID gas: argon (0.0043 mbar); solvent discard: 0–2 and 10–11.5 min.	N.A.	1 mg HCN eq./kg	N.A.	N.A.	2023	[66]
	Linamarin								
	Linustatin								
	Lotaustralin								
	Neolinustatin								
	Prunasin								

BEH: ethylene bridged hybrid; CNG: cyanogenic glycoside; D_2_O: deuterium oxide; ESI: electrospray ionization; H_2_PO_4_: phosphoric acid; HCN: hydrogen cyanide; HLB: hydrophilic–lipophilic balance; HMDS: hexamethyldisilazane; HPLC: high-performance liquid chromatography; HRMS: high-resolution mass spectrometry; HSS: high-strength silica; IMD: methylimidazole; LOD: limit of determination; LOQ: limit of quantification; MRM: multiple reaction monitoring; MS/MS: tandem mass spectrometry; N.A.: data not available; NaCl: sodium chloride; NMR: nuclear magnetic resonance; qNMR: quantitative nuclear magnetic resonance; QqQ-MS/MS: triple-quadrupole mass spectrometry; RRHD: rapid resolution high definition; RSD: relative standard deviation; TMCS: trimethylchlorosilane; TMS: trimethylsilyl; TMSP: trimethylsilylpropionoic acid; UHPLC: ultra-high-performance liquid chromatography. ** BEH is trifunctionally bonded to C_18_. This new technology is versatile, chemical resistant (pH 1–12 and can handle temperatures up to 80 °C), and mechanically stable (can stand high pressures), enhancing retention, specificity, and sensitivity for complex mixtures and a wide variety of compounds [67]. *** This column is stable up to 1200 bar for fast, high-resolution separations of complex samples. It provides good peak shapes for different compounds across a pH of 2–9. The maximum operating temperature is 60 °C [68]. **** High-strength silica particles (made of 100% silica) enhance chromatographic performance by offering increased retention for polar and hydrophobic compounds, providing unique selectivity compared to BEH. It has mechanical strength (pressures up to 1240 bar) and great stability [69].

**Table 2:** Compilation of methods to determine total HCN in flaxseed or flaxseed products.

Sample	Hydrolysis (for HCN Determination)	Extraction	Analytical Technique	LOD	LOQ	Recovery	Precision (RSD)	Year	Ref.
Flaxseed from a previous study, ethanol-extracted ground flax, flax pressed cake, spent flaxseed, and CO_2_-extracted flaxseed.		Extraction of cyanogenic glycosides Extraction solvent: 70% ethanol aqueous solution. Extraction conditions: in a sonic water bath for 30 min at 30 °C. Filtration: with a 0.45 µm filter through glass wool. Extraction of the crude enzyme 1. From ground flaxseed with cold acetone (−20 °C) in a blender for 1 min. 2. Filtration under vacuum through a Whatman No. 1 paper and re-extraction of the residue 3 more times with acetone. 3. Solvent removal on a desiccator under vacuum at 4 °C.	Using a barbituric acid–pyridine reagent: 1. The flaxseed extract was transferred to a stoppered test tube and evaporated with a nitrogen stream. 2. Adding sodium acetate buffer 0.1 M (pH 6) and incubation of the solution with the crude enzyme extract for 1 h at 30 °C. 3. The reaction ends by adding 0.2 M NaOH, then it stands for 5 min at room temperature, and is neutralized with 0.2 M HCl. 4. Chloramine T 0.5% (*w*/*v*) + buffered extract + barbituric acid–pyridine reagent in a test tube that stands at room temperature for 4 min (pink complex). 5. Absorbance measured at 585 nm (using a blank solution). 6. The calibration curve was traced using potassium cyanide as a reference standard (concentration range of 0.1–3.5 µg of HCN).	N.A.	N.A.	94 ± 3%	36.27%	1996 *	[63,64]
	_	4. Extract + cold sodium acetate buffer (pH 6) in a blender for 1 min. 5. Centrifugation: 12,000× *g* for 2 h. 6. The supernatant was put in a fume hood for 3 h at room temperature to allow hydrolysis of any residual cyanogenic glycosides to HCN. 7. The extract was freeze-dried and stored.	Using pyridine–pyrazolone reagent: 1. The flaxseed extract was transferred to a stoppered test tube and evaporated with a nitrogen stream. 2. Adding sodium phosphate buffer 0.1 M (pH 6) and incubation of the solution with the crude enzyme extract for 1 h at 30 °C. 3. The reaction ends by adding 0.1 M NaOH and 0.1 M sodium phosphate buffer (pH 6), to ensure total decomposition of the hydroxynitrites that result from enzymatic activity. 4. Chloramine T 0.5% (*w*/*v*) + buffered extract in a stoppered test tube, mixed, and put in an ice-water bath for 5 min. 5. Adding the pyridine–pyrazolone reagent to the mixture and let the tube stand for 1 h at room temperature (blue complex). 6. Absorbance measured at 620 nm (using a blank solution). 7. The calibration curve was traced using potassium cyanide as a reference standard (concentration range 0.1–3.5 µg of HCN).	N.A.	N.A.		37.37%		[63,64]
Edible plants from 14 generas including *Linum* (flaxseed) from both South and North Korea, and China.	Acid Hydrolysis (50 min) 1. Centrifugation: sample + 0.1 M phosphoric acid for 20 min at 8000 rpm. 2. The supernatant + 4 M sulfuric acid added in a tightly capped vial. 3. Start hydrolysis with heating up to 100 °C. 4. The reaction mixture is cooled in ice.	Distillation Performed in a Micro-Dist tube. 1. The hydrolysis mixture + 0.79 M MgCl_2_ is heated in the tube for 45 min. 2. Cooling at ambient temperature. 3. Cyanide is collected in the 0.2 M NaOH solution.	Ion Chromatography Analytical column: IonPac AS7 (40 mm × 250 mm, 10 μm particle size). Guard column: IonPac AG7 (40 mm × 50 mm, 10 μm particle size). Detector: ED40 (electrochemical detector), DC Amperometry. Flow rate: 1.0 mL/min (isocratic). Injection volume: 200 μL. Electrode cell: silver working electrode (0.00 V vs. Ag/AgCl reference). Mobile phase: 0.5 M sodium acetate/0.1 M sodium hydroxide/0.5% (*v*/*v*) Ethylenediamine.	0.005 mg/L	N.A.	N.A.	<10%	2013	[70]
3 trademarks of each whole flaxseed type: brown and golden. 3 trademarks of bran of each flaxseed type: brown and golden. * All acquired in main supermarkets or natural products stores in Natal/Rio Grande do Norte.	Acid Hydrolysis (3 h) 1. The sample is transferred into a round bottom flask coupled to a distiller. 2. Closing of the distillation system by dipping the condenser’s end in an Erlenmeyer containing 2.5% NaOH solution. 3. Hydrolysis starts by adding distilled water and sulfuric acid 10%.	Distillation 1. Starts when sulfuric acid 10% is added. 2. First distillation: distillate 1 is collected into the Erlenmeyer containing the 2.5% NaOH solution. 3. Second distillation: begins when distilled water and 10% H_2_SO_4_ are added. The distillate 2 is collected in another Erlenmeyer with 2.5% NaOH solution.	Colorimetric Determination Sample tube: an aliquot of distillate + alkaline picrate 0.5% solution (red-orange compound). Blank tube: distilled water + alkaline picrate 0.5% solution. The tubes were shaken, closed, and put in a water bath at 70 °C for 10 min. Calibration curve: for its construction, a sodium cyanide solution (50 µg/mL) was used to prepare 5 standard solutions corresponding to 50, 100, 150, 200, and 250 µg CN^−^. Absorbance was measured at 490 nm.	N.A.	N.A.	N.A.	N.A.	2023	[71]

* These two colorimetric methods were compared to the first HPLC in Table 1. While, in this case, all three methods showed high correlation in measuring cyanogenic compounds, the HPLC method is still the most accurate and sensitive because it can directly distinguish all three cyanogenic compounds. Ag: silver; AgCl: silver chloride; H_2_SO_4_: sulfuric acid; HCl: hydrochloric acid; HCN: hydrogen cyanide; LOD: limit of determination; LOQ: limit of quantification; MgCl_2_: magnesium chloride; N.A.: not available; NaOH: sodium hydroxide; RSD: relative standard deviation.

The UHPLC columns used for these two methods were a C18 RRHD (Rapid Resolution High Definition) (1.8 µm, 2.1 mm × 50 mm) by Zhong et al. and an HSS (High-strength Silica) T3 (trifunctional ligand type) column (2.1 mm × 100 mm, I.D., 1.7 μm) by Cai et al. [59,62]. However, among all the LC methods analyzed, the most used analytical column was C18, made of silica (polar) bonded with octadecylsilane, an 18-carbon chain (non-polar). More specifically a C18 (1.7 µm, 100 × 2.1 mm) column with BEH (ethylene bridged hybrid) particle technology.

### 6.2. Levels of Cyanogenic Glycosides in Flaxseeds

On average, flaxseed contains 250–550 mg of cyanogenic glycosides (CNGs) per 100 g [4]. The Joint FAO/WHO Expert Committee on Food Additives (JECFA) has suggested that acute symptoms of cyanide toxicity, such as vomiting, nausea, headache, mental confusion, hyperpnea, and a decrease in blood pressure, can occur at doses exceeding this range. JECFA has established a safe daily intake of cyanide at 90 µg per kilogram of body weight [71,72], while toxic levels for humans are reported to be between 30 and 100 mg per day [70]. The diary flaxseed consumption of 50 mg showed no adverse effects on human health, which happens because the adult human body is able to detoxify ≤100 mg cyanide/day, and food processing like cooking can reduce the content of these compounds [26].

The EFSA (The European Food Safety Authority) Panel on Contaminants in the Food Chain (CONTAM) issued a scientific opinion, concluding that the acute reference dose (ARfD) for cyanide established at 20 µg per kg of body weight in raw apricot kernels is applicable, as it is considered protective against acute cyanide effects, regardless of the dietary source [73]. Even though setting different ARfDs for different food types is not considered appropriate, this value may be overly conservative due to the lower bioavailability of cyanide from flaxseed, which led to the establishment of a bioavailability factor of 3 [73]. Flaxseed was one of the foods with the highest mean cyanide concentration reported (192.1 mg/kg) in the EFSA database; however, there are no reports in the literature of human poisonings due to the consumption of flaxseed. Using the highest cyanide value reported in the EFSA database (407 mg of CN^−^/kg) as a worst-case scenario, the maximum amount of ground flaxseed that can be consumed without exceeding the ARfD ranges from 1.3 g to 14.7 g, depending on body weight. The ARfD would be exceeded in a toddler with a consumption of about 4 g (almost a teaspoon). Due to all uncertainties, some risks remain for adolescents if they consume ground flaxseed [73]. Care is needed with foods like ground flaxseed, particularly for sensitive groups like toddlers and adolescents, and the consumption of intact flaxseed would likely result in much lower cyanide exposures. It was also concluded that the primary contributors to exposure were biscuits, juice or nectar, and pastries and cakes [73]. This report by CONTAM also presents the acute lethal oral dose of cyanide in humans that is estimated to be between 0.5 and 3.5 mg/kg of body weight [73].

The CN- reported by Cho et al. ranged from 261.9 to 345.4 µg/g, which is in the same range as the one reported by Pereira et al., which was 348.40–467.54 µg/g [70,71]. The values for linustatin and neolinustatin registered, respectively, were 1.37 mg/g and 1.55 mg/g according to Zhao et al., 2.0–5.7 mg/g and 0.9–3.9 mg/g according to Shawar et al., and 0.22–2.83 mg/g and 0.29–3.65 mg/g according to Zhong et al. [59,60,65]. The study by Pereira et al. also calculated the equivalent of the suggested safe daily intake, which corresponded to two full tablespoons of whole seeds per day or one full tablespoon of bran per day should be the maximum quantity consumed, which shows the importance of adding the total CN- content in commercial packages to ensure the consumption of safe and nutritious food [71]. Also, in the same study, significant differences were found between whole flaxseed and flaxseed bran, but not between brown and golden types.

The European Union, concerned about the presence of cyanogenic glycosides, has established regulatory limits to ensure food safety (Commission Regulation EU 2023/915 of 25 April 2023). The maximum content of hydrogen cyanide or hydrogen cyanide in cyanogenic glycosides in flaxseed. For unprocessed flaxseeds, whole, crushed, ground, split, and chopped are not placed on the market for the final consumer, and 250 mg/kg is the maximum content allowed to be present. For the ones placed on the market for the final consumer, 150 mg/kg is the higher value of this contaminant that can be present in flaxseed [74].

The Rapid Alert System for Food and Feed (RASFF) is a crucial tool used by the European Union to ensure food safety across its member states. It allows for the rapid exchange of information about potential risks to human health posed by food, feed, and food-contact materials, including alerts [75]. Through RASFF, EU countries, along with the European Commission, can rapidly coordinate responses, such as product recalls or import rejections, to protect consumers. The purpose of this tool is to enhance transparency and consumer protection and maintain high food safety standards across Europe [75]. From a quick search on the RASFF window notification of alerts referring to cyanogenic compounds on flaxseed, five recent notifications were found. In May 2022, there was an alert on increased cyanide content (280 ± 77 mg/kg) in organic flaxseed from Germany, by official controls on the market. Many European organizations were involved, and the measures taken by Germany were withdrawal from the market, informing recipient(s), and destruction [76]. In February 2023, there was an alert on hydrogen cyanide in organic blond flaxseed from Turkey (300 ± 60 mg/kg), based on the company’s own test. The recipient(s) were informed (by the Netherlands) and destruction also took place (by Romania) [77]. In April 2024, Denmark notified others of the presence of hydrogen cyanide in flaxseed (320 mg/kg) from Poland and hydrogen cyanide in flaxseed from Belgium (340 mg/kg), both resulting from an official control of the market and involving other European countries on the follow-up [78,79]. The product was withdrawn from the market (by Denmark), and measurements were taken related to the first notification [78]. Lastly, in the same month, Denmark alerted others to the presence of hydrogen cyanide in organic brown flaxseed from India (320 mg/kg) following another official control on the market. The product was then withdrawn from the market [80]. Fortunately, over the past two years, there have been only five notifications, all of which were resolved quickly without any harm to the consumer. The goal is to further reduce the number of such issues and to detect these occurrences as early as possible. Identifying these elevated, undesirable levels is crucial to prevent disastrous events. Therefore, the development and enhancement of detection techniques and tools like communication channels are clearly both useful and essential. Detoxification methods are as valuable to the purposes of protecting the consumer as the other resources referred to before. Some of these methods are described below.

### 6.3. Detoxification Methods

**Figure 3:**
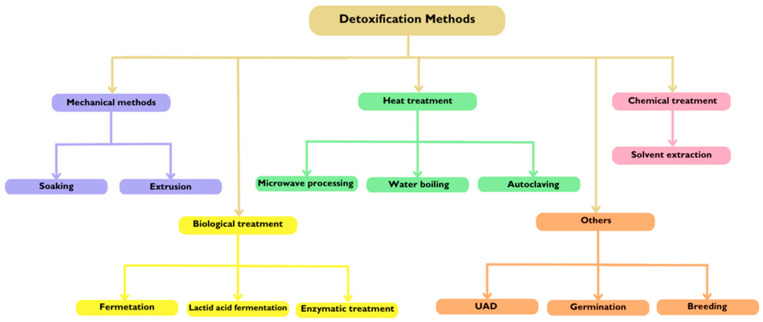
Detoxification methods to reduce the content of flaxseeds in cyanogenic glycosides. UAD: ultrasound-assisted detoxification.

It is crucial to ensure the removal or reduction of these antinutritional compounds. Cyanogenic glycosides can be significantly reduced through food processing methods, both on a large scale in the food industry and on a smaller scale with simple household techniques. The food processing industry can develop new methods or improve existing ones to effectively control cyanogenic glycoside levels, ensuring the safety and nutritional quality of flaxseed products. In Table 3 there are some studied and detailed methods for these purposes (Figure 3).

The use of microwaves and boiling water in cooking, common daily practices, can be effective allies in removing cyanogenic glycosides from flaxseed [81]. Soaking, although the least effective method, still contributes to safer flaxseed consumption [81].

**Table 3:** Compilation of detoxification methods to remove cyanogenic compounds in flaxseed or flaxseed products.

Sample	Detoxification Technique	Description	Removal Rate	Study Conclusion	Year	Ref.
Flaxseed, Nei Ya-3 Cultivar, northeast China	Extrusion	Equipment: co-rotating twin-screw extruder; length-to-diameter ratio of 27.9:1; screw diameter of 47 mm; circular die of 5.2 mm; equipped with two heating units. Optimal conditions obtained: temperature: 146.0 °C; feeding rate: 32.7 kg/h; crew speed: 152.5 rpm; moisture content: 12.5%.	91.62%	The predicted value for the removal of HCN from flaxseed was 93.23% and the experimental result was 91.62%, constituting a relative error of only 1.76%. The feeding rate was increased to 60 kg/h to improve productivity. The experimental value was 83.32% (relative error of 1.27%). Both detoxification levels were within the required limits.	2008	[82]
Fresh flaxseed from the Lanxi County of Heilongjiang Province, China	Enzymatic treatment/fermentation	Enzymatic preparation: 12.5% human liver ꞵ-glucosidase and 8.9% (*w*/*w*) *Bacillus* sp. cyanide hydratase prepared in the laboratory. Standard fermentation medium: flaxseed power + water + MgCl_2_ + MnCl_2_; pH adjusted to 6.3; autoclaved at 115 °C for 1 h to inactivate the endogenous β-glucosidase. The fermentation medium and the enzymatic preparation were mixed and incubated at 46.8 °C for 48 h. After that, the residual cyanide and the CNGs were measured in the samples.	99.3% (degradability)	The flaxseed power treated with the enzymatic preparation retained lignans and fatty acids. Although this method seems efficient, low-cost, and a protector of beneficial nutrients, it is necessary to prove that the detoxified flaxseed is safe for consumption. This method also provides new ways of removing CNGs from other edible plants.	2012	[83]
Flaxseed from 3 Polish high-α-linolenate flax varieties: 1 brown (Szafir variety) and 2 golden seeds (Oliwin and Jantarol varieties).	Solvent extraction method	Defatting previous double cold extraction with hexane. Aqueous extraction: DFF to water ratio of 1:15 (*m*/*v*), under constant stirring with a magnetic stirrer at ambient temperature for 1 h. Centrifugation: for 25 min, 1500× *g*. The supernatant was freeze-dried.	95.7–97.7%	This was a comparative study of flaxseed extracts since they can be applied as food ingredients. Aqueous extracts demonstrated a significant reduction in CNGs but less antioxidant activity. Ethanolic extraction, although it resulted in higher antioxidant activity, also showed a higher CNG content, and if used, ethanolic extracts have to be controlled. For detoxifying purposes, flaxseed processing with water such as extraction, but also soaking and wet autoclaving, can decrease significantly CNG content.	2015	[84]
		Ethanolic extraction: DFF was extracted twice with 60% aqueous ethanol with a flaxseed to solvent ratio of 1:7.5 (*m*/*v*), under constant vigorous shaking using a lab-scale orbital, at ambient temperature for 1 h. Filtration; centrifugation: 20 min, 1500× *g*. Removal of ethanol by evaporation of the supernatant using a rotary vacuum evaporator. The samples were freeze-dried.	30.2–33.6%			
5 samples of flaxseed intended for use in animal feed	Enzymatic hydrolysis followed by evaporation	Mixing, grinding, and extraction (with an acidic aqueous solution): adding one or more cellulose or starch materials (wheat bran and sunflower cake) helps disrupt the cells and facilitates the interaction between ꞵ-glycosidases (short-term vapor impregnation for activation) and the CNGs. The maturation: provides sufficient time for the hydrolytic reaction (pH 6 and 38 °C). Extrusion applies pressure and temperature that reduce the HCN and water content. Drying and cooling: decrease the humidity and increase the stability of the final detoxified material (most of the HCN is evaporated during the physical part).	89–92%	The levels of hydrogen cyanide (HCN) in the 5 samples ranged from 86 to 127 mg/kg, which were reduced to <11 mg/kg after decontamination. An additional study on decontaminated samples containing flaxseed from various batches showed an average HCN content of 12 mg/kg. The method proved efficacy, meeting EU requirements for the quality of flaxseed concerning HCN after decontamination, without affecting the nutritional quality or leaving harmful residues. Emissions of HCN into the atmosphere must comply with national regulations.	2017	[85]
Brown and golden flaxseed	Germination	This method was carried out for 5 days in a dark and closed incubator at 25 °C. To avoid microbial growth seeds were washed twice a day and collected daily to measure the changes in root length. The samples were treated and oven-dried at 105 °C to constant weight to determine their content. The results correspond to the percentage of weight loss during drying.	Linustatin: 1309.09 ± 68.63 μg/g to 112.73 ± 7.44 μg/g (≈91.39%). Neolinustatin: 1144.91 ± 22.82 μg/g to 93.89 ± 5.77 μg/g (≈91.80%). Lotaustralin: 1138.46 ± 20.29 μg/g to 72.99 ± 7.26 μg/g (≈93.59%).	Even though germination significantly increased linamarin content, it reduced the other antinutritional compounds. This method also promotes active components, increasing SDG content, flaxseed oil, phenolic and vitamin E content, and thus flaxseed antioxidant activity.	2019	[86]
Flaxseed mixture of brown and golden seed varieties from local market in Beijing	Microwave processing	Samples were dispersed on glass plates. Equipment: household microwave oven. Frequency: 2450 MHz. Output power: 450 W. Heating up time: 12 min.	70.9%	The water-boiling method eliminated the highest CNG content followed by UAD, solvent extraction method, microwave processing, autoclaving, and soaking. However, the analysis of this ranking needs to consider the disadvantages of the different methods. The water-boiling method requires a high temperature that may affect the functional properties of flaxseed since it causes vitamin loss and amino acid and protein denaturation. UAD, in turn, can be an effective method and not affect the beneficial nutrients under controlled conditions.	2020	[81]
	Water-boiling method	DFF was poured into boiling water (100 °C) for 30 min with continuous stirring, then cooled down at room temperature, centrifuged (3000 rpm, 15 min, 25 °C), filtered, and freeze-dried.	94.3%			
	Autoclaving	Equipment: autoclave machine. Temperature: 120 °C. Time: 20 min.	62.6%			
	Ultrasound-assisted detoxification (UAD)	The samples were mixed with distilled water in a solid–liquid ratio of 1:20 (*w*/*v*) and stirred in a water bath for 30 min. Equipment: Sonics Vibracell Probe. Power: 300 W; frequency: 20 kHz; pulsed mode: 10 s ON and 3 s OFF; temperature: 50 °C; time interval: 20 min.	92.1%			
	Soaking	DFF was soaked in distilled water with a continuous change in water. Time interval: 48 h. Samples were submitted to a vacuum filtration and freeze-dried.	48.2%			
	Solvent extraction method	Extraction solvent: methanol/ammonia/water (90/5/5). The mixture was stirred (500 rpm) for 15 min, vacuum filtered, and freeze-dried. The extraction was repeated twice for each sample.	82.4%			
Whole sorrel flaxseed from G.A & Robin Fenton Farm and G&G Edmunds Farm	Bench-scale fermentation	Equipment: square narrow-mouth polypropylene fermentation bottle with a gas trap. Inoculation of a mixed culture of *Lactobacillaceae* (i.e., *Lactobacillus* sp., *Limosilactobacillus* sp., and *Lactiplantibacillus* sp., *Lacticaseibacillus* sp., *Levilactobacillus* sp., and *Lentilactobacillus* sp.) in-house cultured previously isolated from wheat-based thin stillage in a solution containing whole flaxseed + commercial white vinegar (3% acetic acid *v*/*v*; pH ≈ 3) + distilled water. Conditions of incubation: water bath at 30 °C for 72 h. Preparation for analysis: the fermentation media was passed through a 12′′ 200 µm test sieve, rinsed with tap water, dried overnight at 60 °C, and ground in a coffee grinder.	After 48 h of fermentation, linustatin and neolinustatin were below the detection limits and, consequently, total HCN < 10 mg/kg.	Fermentation of flaxseed using *Lactobacillaceae* successfully removed cyanogenic glycosides and retained beneficial nutritional components such as oil, fatty acids, and SDG. The proposed method can be readily implemented in flaxseed processing based on a pilot-scale feasibility study but lacks further development to produce higher quality flaxseed that can be safely and readily used in food and other product production.	2023	[87]
	Scale-up fermentation	These experiments were conducted at 2 volumes, 4 L and 8 L: sorrel flaxseed and bacterial inoculant. Fermentation took place in a mechanical oven incubator at 30 °C for 72 h. Flaxseed was degummed after being rinsed with tap water and dried, spread in silicone sheets, using a 160 L commercial-grade food dehydrator at 60 °C.	Linustatin: 2659.3 ± 45.7 mg/kg to <500 mg/kg (>81.2%). Neolinustatin: 2761.4 ± 16.8 mg/kg to <500 mg/kg (>81.9%). Total HCN: 351.8 ± 2.5 mg/kg to <10 mg/kg (>97.2%).			

CNG: cyanogenic glycoside; DFF: defatted flaxseed flour; MgCl_2_: magnesium chloride; MnCl_2_: manganese chloride; SDG: secoisolariciresinol diglycoside; UAD: ultrasound-assisted detoxification.

Another mechanical method is extrusion and it also showed a desirable removal rate [82].

Solvent extraction is another viable option, provided the right solvent or mixture is used, though it may also remove valuable constituents [81,84]. Heat treatments, such as water boiling (with the highest removal rate of the physical methods) and autoclaving, can cause protein denaturation and vitamin loss. To mitigate these drawbacks, new techniques have been studied and developed, such as ultrasound-assisted extraction [81], enzymatic treatment, fermentation [83,87], and germination [86]. These methods show promising removal rates and potential for large-scale application. However, some of these methods will require biological control [87].

Instead of processing flaxseed to reduce these compound levels, it is possible to produce seeds with an already low content of cyanogenic glycosides through breeding. A study by Russo & Reggiani on this matter focused specifically on the respective content of these antinutrients in 21 varieties of flaxseed that belonged to 3 different groups of productive attitude: oil, intermediate, and fiber. The intermediate group showed the lowest linustatin content, with the variety Festival standing out for having the lowest total CNG content (0.74 g/kg CN-) and the lowest linustatin content (0.28 g/kg CN-) among all varieties. In the fiber group, the lowest neolinustatin content was observed compared to the other groups, along with varieties that showed low levels of CNG content. In the oil group, the variety Ita269 had the highest CNG content (1.60 g/kg CN-), while the variety Ventimiglia had the lowest neolinustatin content of all varieties. The variability in CNG content across these groups can be explored in breeding programs aimed at reducing CNG levels in flaxseed varieties [88]. However, there are other studies that, apart from evaluating the lower levels of these compounds, also evaluated the differences between compositions to choose the variety that is safer and has more nutritional value. A study by Pavelek et al. evaluated the results of flaxseed breeding in the Czech Republic, which are two recently released Czech varieties and a new breeding line registered for growing that year. The first ones were compared to a standard Dutch variety but had different fatty acid compositions. The last one represents a new quality type given by the content of both linoleic and linolenic fatty acids and the low content of cyanogenic glycosides [89]. A study by Salem et al. applied a comprehensive metabolic and lipidomics approach to the selection of flaxseed varieties. This study evaluated six flaxseed cultivars from different localities in Egypt, identifying Sakha 6 as the most promising. These yellow-colored seeds showed a high content of key amino acids, the highest of two essential PUFAs, α-linolenic acid and linoleic acid, and medicinally important secondary (like riboflavin) metabolites content was higher in this cultivar, while presenting low levels of undesirable antinutrients, such as cyanogenic glycosides. Both the results from breeding and this more recent study suggest that specific flaxseed cultivars are especially beneficial for human consumption and provide valuable data for selecting and developing flaxseed varieties with more quality in the future [90].

Each method has its advantages and disadvantages and may require ongoing refinement. However, it is essential to choose techniques that maximize the removal of cyanogenic glycosides while preserving the beneficial and nutritional components of flaxseed, ensuring its safe consumption.

## 7. Conclusions

Flaxseed stands out as a highly beneficial dietary component, being a rich source of omega-3 fatty acids, lignans, proteins, and fiber, which play significant roles in cardiovascular health, by reducing blood pressure and positively impacting the lipid profile, cancer prevention, through lignans that exhibit antioxidant properties, and anticancer effects, improving tumor biological markers, diabetes management, by enhancing glycemic control and insulin sensitivity, and gastrointestinal health, working as a prebiotic or increasing the fiber intake, which is very important in managing constipation. Not only does flaxseed help prevent various health issues, but it also enhances the quality of life for healthy individuals. While incorporating flaxseed into the diet has demonstrated promising benefits in these areas, it is not sufficient on its own. With the increasing interest in this nutraceutical, it is crucial to continually explore and deepen our understanding of its health impacts.

However, despite its benefits, this article also underlines potential risks associated with flaxseed consumption. The presence of phytic acid and trypsin inhibitors, but, specially, of cyanogenic glycosides, poses challenges that must be addressed through proper processing and controlled intake. Advances in analytical techniques have significantly improved the extraction, detection, and quantification of these last antinutrients. Techniques such as ultra-high-performance liquid chromatography–tandem mass spectrometry (UHPLC-MS/MS) and quantitative nuclear magnetic resonance (qNMR) have demonstrated high sensitivity, precision, and reliability in identifying and quantifying cyanogenic glycosides.

To ensure consumer safety, a variety of detoxification methods have been explored, ranging from mechanical and biological treatments to heat and chemical approaches. Additionally, innovative techniques such as ultrasound-assisted detoxification and advanced breeding strategies have shown promising removal rates for contaminants like cyanogenic glycosides. Notably, biological or enzymatic treatments present significant potential for large-scale application while minimizing nutrient loss often associated with heat treatments, maintaining the overall nutritional value of flaxseed.

In conclusion, ongoing research, coupled with technological advancements and genetic improvements, is vital for enhancing detection and processing methods for contaminants. Future studies focused on the complex interactions between flaxseed components and various medications will be essential for ensuring their safe integration into diverse dietary regimens. Equally important is the investigation of recommended dosages, which must be tailored according to individual factors such as specific health conditions, medical history, age, and gender. As science continues to advance, flaxseed can be confidently embraced not only as a nutritious and health-promoting addition to diets but also as a secure option for consumers seeking to optimize their overall well-being. By harnessing these innovations and insights, we can unlock the full potential of flaxseed while prioritizing safety and efficacy in its consumption.

## Data Availability

Data sharing is not applicable to this article.
